# Nanoparticles as a younger member of the trace element species family — a food perspective

**DOI:** 10.1007/s00216-023-04940-z

**Published:** 2023-09-15

**Authors:** Zuzana Gajdosechova, Katrin Loeschner

**Affiliations:** 1 National Research Council Canada, Metrology, 1200 Montreal Road, Ottawa, ON K1A 0R6 Canada; 2 Technical University of Denmark, National Food Institute, Kemitorvet 201, 2800 Kgs. Lyngby, Denmark

**Keywords:** Nanoparticles, Speciation, Food, Gastrointestinal tract, Dissolution, Biogenic

## Abstract

Speciation analysis plays a key role in understanding the biological activity and toxicity of an element. So far, classical speciation analysis focused only on the dissolved fraction of an elemental species, whereas nanoparticle forms of analytes are being widely found in consumer and industrial products. A significant contributor to human exposure to nanoparticles is through food into which nanoparticles can be incorporated from endogenous sources or they may be formed naturally in the living organisms. Nanoparticles often undergo changes in the food matrices and upon consumption, in the gastrointestinal tract, which present a significant challenge to their characterisation. Therefore, a combination of both classical and nanoparticle speciation analytical techniques is needed for the characterisation of both dissolved and particulate forms of the chemical species. This article presents and discusses the current trends in analysis of nanoparticle behaviour in the gastrointestinal tract and formation and characterisation of biogenic nanoparticles.

**Graphical Abstract:**

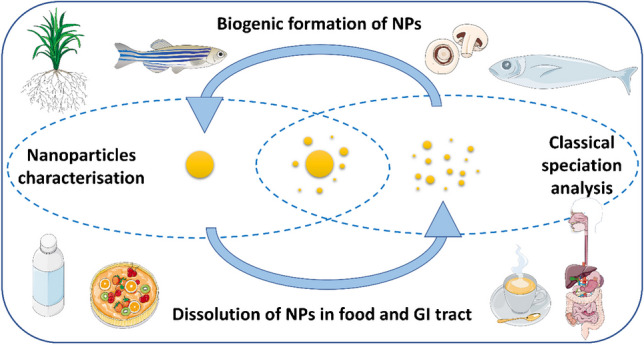

## Introduction

The International Union of Pure and Applied Chemistry (IUPAC) defines chemical species as “specific form of an element defined as to isotopic composition, electronic or oxidation state, and/or complex or molecular structure” [1]. The group of trace elements that has been extensively studied in relation to their chemical species in food included arsenic, mercury and selenium [2] and is continuously expanding to reflect current and anticipated regulatory needs. The speciation of elements in food is key in understanding their uptake, biological activities and toxicity [3]. The classical speciation analysis requires typically two steps: (1) separation of the different chemical forms and (2) detection and quantification of each chemical form. The often used techniques are high-performance liquid chromatography (HPLC) or gas chromatography (GC) coupled to mass spectrometry (MS) or inductively coupled plasma mass spectrometry (ICP-MS). Confirmation and identification of unknown species are achieved by molecular mass spectrometry or other structure‐characterising techniques.

**Fig. 1:**
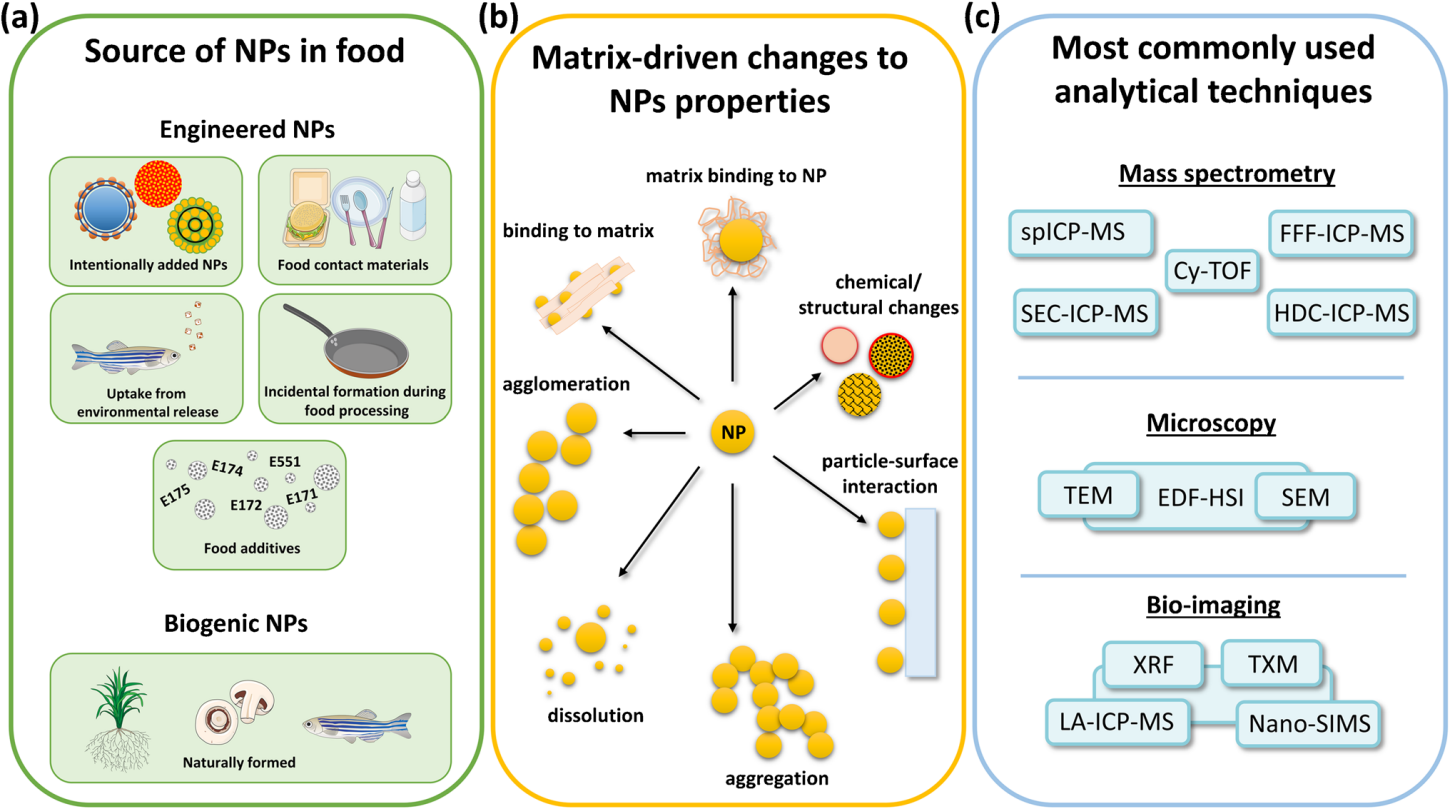
An overview of NP sources in food (**a**), matrix-driven changes to NP properties (**b**), and most used techniques for NP analysis (**c**) (spICP-MS, single-particle inductively coupled plasma mass spectrometry; FFF-ICP-MS, field flow fractionation ICP-MS; SEC–ICP–MS, size exclusion chromatography ICP-MS; HDC-ICP-MS, hydrodynamic chromatography ICP-MS; Cy-TOF, cytometry time of flight; TEM, transmission electron microscopy; SEM, scanning electron microscopy; EDF-HSI, enhanced dark-field hyperspectral imaging; XRF, X-ray fluorescence; TXM, transmission X-ray microscopy; LA-ICP-MS, laser ablation ICP-MS; nanoSIMS, nanoscale secondary ion mass spectrometry)

Nanoparticles (NPs) are discrete pieces of material with at least one dimension in the size range of 1 to 100 nm. Inorganic NPs can be considered a distinct chemical species of elements themselves. Engineered nanomaterials are nowadays widely integrated into consumer and industrial products. Besides cosmetic and cleaning products, food is a major source of exposure of consumers to NPs through ingestion [4]. Recent year’s research has shown that the origin of inorganic NPs in the food can be diverse. In most instances, engineered NPs are intentionally added into the food or are present as food additives containing a fraction of particles at the nanoscale size, but they also can be released from food contact materials (Fig. 1a). Additionally, engineered NPs unintentionally released to the environment can re-enter the food chain, and in vivo formation of NPs was also reported.

NPs have been intensely studied throughout the last two decades with regard to their synthesis, applications and toxic effects. Alongside, qualitative and/or quantitative analytical techniques for characterisation of NPs have been developed or adapted. Among these techniques are single-particle ICP-MS and ICP-MS coupled to separation techniques like field flow fractionation (FFF), capillary electrophoresis (CE), hydrodynamic chromatography (HDC) and reversed phase HPLC (Fig. 1c). Bio-imaging techniques such as laser ablation ICP-MS, nanoscale secondary ion mass spectrometry (nano-SIMS) and X-ray fluorescence support the qualitative analysis of NPs, whereas electron microscopy is typically considered the gold standard when it comes to determination of particle sizes. Electron microscopy provides additional information on particle shape, crystal structure and if combined with spectroscopy, chemical composition can be determined. One major analytical challenge is the sample preparation as NPs can agglomerate, aggregate, dissolve or undergo changes of their crystalline structure/chemical composition as response to new surroundings (Fig. 1b). They can further interact with surfaces (of, e.g. storage containers and analytical equipment) and matrix components like fibres or proteins [5].

Like other elemental species, NPs might undergo changes in the food matrices or ultimately in the human gastrointestinal tract (GIT). The changes occurring to NPs are the same as described above for sample preparation (Fig. 1b). Dissolution of NPs is especially of interest for human risk assessment, as complete dissolution in either the food matrix or the GIT would imply that no nano-specific considerations will be required. It is then expected that the NPs behave like their corresponding soluble form. The released ions might also form new organic or inorganic species which can be studied by classical speciation analysis.

Another interesting research area, where NP characterisation and classical speciation analysis meet, is the biogenic formation of NPs (also called in vivo formation or naturally formed NPs), i.e. particles that are formed inside the living organism. Understanding the mechanisms and reasons for their formation requires both characterisation of the species involved in the formation of the NPs and the NPs themselves. This often needs combination of several analytical techniques due to the complexity of biogenic NPs and the matrix in which they are present. This trend article describes the current status of analytical work in the research area of NP dissolution in food and the GIT as well as the in vivo formation of NPs in food-relevant systems to highlight the important role of NPs as a member of the trace element species family and the link to classical speciation analysis. It is out of the scope of this article to provide a comprehensive review and thus where relevant the readers are directed to published review papers for more details. Finally, we provide our views on the future developments and research directions that will lead to improvement of NP analysis and their amalgamation into the world of trace element speciation.

## Dissolution of NPs in the GIT and food

It is known that NPs can change and interact with their surroundings, something that will also happen in a food matrix and the gastrointestinal environment. This includes, e.g. binding of matrix constituents to the NPs (protein corona), agglomeration/deagglomeration of the NPs and NP dissolution. Dissolution is particularly interesting as fast dissolution of the NPs in water, food or the GIT would imply that no intact NPs will be adsorbed by humans and consequently no specific assessment of the risk related to the nanoscale is required, i.e. no in vitro and in vivo testing of the NPs [6]. Further, dissolution kinetics of NPs is considered a key factor that determines their biopersistence and biodurability in different biological systems [7], although it is so far unknown how biodurability and persistence or lack thereof for a particular nanomaterial affect the overall gut health chronically.

Dissolution of NPs is besides their intrinsic properties (e.g. size, chemical composition, surface properties) affected by the surrounding media [8]. A popular way of studying the dissolution of NPs is static in vitro digestion models that simulate human oral digestion, accounting for salt and protein composition, pH changes, transit times and volume changes, which occur in the GIT during food passage. They are typically based on solubility assays that are used for determining the bioaccessibility of certain compounds, e.g. toxic elements and mycotoxins, from food. Table 1 presents examples of in vitro digestion studies that determined the dissolution of NPs. Typically, at least two stages of GIT (stomach and intestine) were simulated. Several studies included the mouth stage where food is combined with artificial saliva. Sometimes the influence of a food matrix was investigated.

**Table 1:** Examples of static in vitro digestion studies experimentally determining the dissolved fraction of NPs and analytical approaches that were applied to determine the dissolved fraction

Studied NPs	Number of stages	Organic components in digestion fluids (* = proteins/protein-containing)	Influence of food matrix studied	Separation of dissolved fraction	Analysis of dissolved fraction	Ref
Ag and TiO_2_	2 (stomach pH 2, intestine pH 7)	Bile salts, pancreatin*, pepsin*	Yes: molluscs and surimi	No	spICP-MS	[9]
TiO_2_, SiO_2_, ZnO, Fe_2_O_3_	3 (mouth pH 6.5, stomach pH 1.4, intestine pH 8.1)	Amylase*, bile*, bovine serum albumin*, glucosamine-hydrochloride, glucose, glucuronic acid, lipase*, mucin*, pancreatin*, pepsin*, urea, uric acid	No	Filtration (0.02-μm syringe filter)	SF-ICP-MS	[7]
Ag	3 (mouth pH 6.8, stomach pH 2.5, small intestine pH 7.0)	Amylase*, bile salts, mucin*, pancreatin*, pepsin*	No	Ultrafiltration (3 kDa)	ICP-MS	[10]
Fe_2_O_3_	3 (mouth pH 6.8, stomach pH 2, small intestine pH 7.0)	Amylase*, bile salts, lipase*, mucin*, pepsin*	Yes: 2% corn oil-in-water emulsion (97.8 wt% phosphate buffer) stabilized with 0.2 wt% whey protein (model food)	Centrifugation (19,357 × g, 4 h)	ICP-MS	[11]
Ag	3 (mouth pH 6.8, stomach pH 2.5, small intestine pH 6.5)	Same as in [7]	No	Ultrafiltration (3 kDa)	ICP-AES	[12]
Al^0^, γAl2O3	3 (mouth pH 6.4, stomach pH 2, intestine pH 7.5)	Amylase*, bile*, mucin*, pancreatin*, pepsin*, trypsin*, urea, uric acid	No	Ultracentrifugation (100,000 × g, 1 h)	ICP-MS	[13]
Ag	3 (mouth pH 6.4, stomach pH 2, intestine pH 7.5)	Same as in [13]	Yes: equal portions of milk powder, starch and olive oil (representing main food components proteins, carbohydrates, fatty acids)	Ultracentrifugation (100,000 × g, 1 h)	AAS	[14]
CdSe (core)/ZnS (shell) quantum dots	2 (stomach pH 2, intestine pH 7.5)	Bile salts, mucin*, pancreatin*, pepsin*, urea	No	Centrifugal ultrafiltration (10 kDa, pore diameter 2.8 nm)	ICP-OES	[15]

*spICP-MS* single-particle ICP-MS, *SF-ICP-MS* sector field ICP-MS, *ICP-AES* inductively coupled plasma atomic emission spectrometry, *AAS* atomic absorption spectrometry, *ICP-OES* inductively coupled plasma optical emission spectrometry

The separation of the dissolved fraction was typically achieved by (ultra)filtration or (ultra)centrifugation. The dissolved fraction was consecutively analysed by an elemental analysis technique. It should be considered that the obtained “dissolved fractions” can contain free ions and/or small organic and inorganic complexes, whereas the retained fraction might not only contain NPs but also matrix-bound ions (e.g. ions bound to proteins, salt precipitates). Bove et al. [12] performed control experiments with standard Ag^+^ solution in the in vitro dissolution test and showed that 82% was retained by the filter. The data suggested that Ag ions, after being released from NPs, interact with the matrix through various processes, including Ag chloride formation or chelation to sulphur groups of proteins. However, no further speciation analysis was attempted. Wu et al. [10] calculated the concentration of the “Ag-biomolecules” formed in the gastrointestinal fluids by collecting the material retained by the filter during ultrafiltration and determining the fraction of Ag from Ag chloride (based on Cl^−^ analysis with ion chromatography) in relation to the total Ag concentration.

Few techniques allow both the characterisations of NPs and ionic species. Single-particle ICP-MS is claimed to provide information about particle size distribution and dissolved fraction at the same time and has been applied to study the behaviour of silver and TiO_2_ NPs in an in vitro digestion model [9]. One of the limitations of the spICP-MS technique is that it cannot distinguish between ions, small organic/inorganic complexes and small NPs (i.e. NPs below the size limit of detection). Validation studies that assess the accuracy of dissolved concentrations, especially in complex matrices, are lacking. Mass and number concentrations determined by spICP-MS are known to have limited accuracy in complex samples [16].

**Fig. 2:**
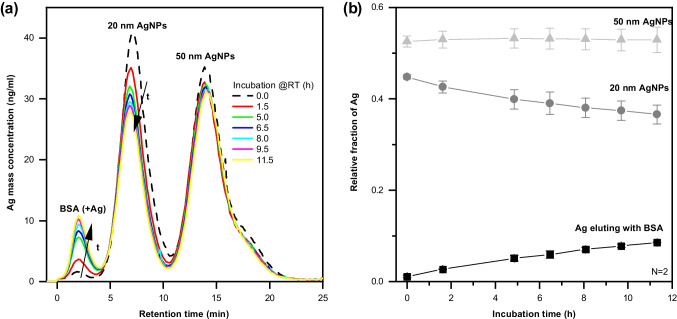
Investigation of the dissolution of 20 and 50 nm Ag NPs spiked to bovine serum albumin (BSA) solution at room temperature by AF4-ICP-MS: **a** fractograms based on quantified ICP-MS signal, **b** relative fraction of Ag in NP form and bound to BSA (unpublished results)

An alternative for studying NPs and their dissolution products in complex systems such as GIT fluids might be asymmetric flow-field flow fractionation (AF4) coupled to ICP-MS. It can provide information about NPs and small complexes and is suitable for larger NPs (up to approximately 1 µm). Figure 2 shows an example where the dissolution of silver NPs in bovine serum albumin at room temperature was studied by AF4-ICP-MS. The technique was further used by Bolea et al. to study silver NPs and dissolved species of silver in culture media and cells [17]. UV–Vis absorption spectrometry was applied to provide information about the nature (organic vs. NP) of the eluted species, and silver was monitored by ICP-MS. The release of silver ions from the NPs and their association with the proteins present in the culture medium were observed. It should be mentioned that the characterisation of the NPs and the protein-bound silver required two separate runs using AF4 with different cross flow programs. AF4-ICP-MS was used to investigate selenium NPs incubated in gastrointestinal conditions [18], but no dissolution was observed.

The disadvantage of AF4 for studying dissolution is usually that free ions and small complexes can pass through the AF4 membrane (3–30 kDa) with the applied cross flow. Collection of fractions of the cross flow for obtaining information on the dissolved portion of the analyte is rarely done due to the delayed elution through the frit of the channel, dilution and elevated blank levels for some elements [19]. Therefore, Tan et al. developed an approach that allowed online monitoring of the dissolved fraction in the cross flow [19]. This was applied to determine the dissolved fraction in environmental water samples and validated by comparison with an ultrafiltration-based approach.

Another technique for investigating NP dissolution which is also based on an online separation coupled to ICP-MS is sodium dodecyl sulphate (SDS)-containing reversed phase liquid chromatography. This technique provides information about the NPs, small complexes and ions in a single chromatographic run. The limitation is the upper size range of 50–60 nm of NPs that can be studied [20]. The technique has not been applied in the context of in vitro digestion of NPs yet but has been used for the speciation of gold NPs and low-molecular gold species in Wistar rat tissues [21]. It was further used to study the uptake of iron oxide NPs (iron supplement) by Caco-2 cells. The presence of three different fractions corresponding to NPs aggregates, dispersed NPs and soluble iron was observed [20].

In summary, dissolution of NPs in GIT has been mainly assessed with simple analytical approaches. The application and development of advanced characterisation techniques are required to quantify “matrix-bound” and “free” ions to avoid an underestimation of the NP dissolution. Inorganic or organic complexes as products of the dissolution should be correctly identified as they might have a different biological activity than the free ions. Classical speciation techniques could play an important role in these tasks.

Few studies on NP dissolution in food matrices and food simulants exist. The dissolution of silver NPs was studied in acidic, alcoholic and fatty food simulants [22]. AgNPs behaved in an example of a real food (low fat milk) in the same way as in the corresponding food simulant (ethanol 50%). However, it was discussed that this might not be true for all other foods as it was, e.g. shown that salt (NaCl) which is present in some foods but not in the food simulants enhanced dissolution under migration conditions. The percentages of dissolved Ag determined by spICP-MS were higher than by ultrafiltration followed by ICP-MS analysis. This was explained with the different “size cut-offs” of the two methods: spICP-MS considers also NPs below size LOD (20 nm in the study) as “dissolved” Ag, whereas ultrafiltration uses a filter with a molecular cut-off corresponding to NPs of a few nanometres in size.

## Formation and characterisation of biogenic NPs

The triggers leading to formation of biogenic NPs are not fully understood, and it can be assumed that they are element specific. In some instances, it could be a result of detoxification mechanism, whereas in others, a deposition of an excess element. The actual location of biogenic NPs can be intra- or extracellular, and very often, they have multi-elemental composition. Biogenic NPs have been found in various shapes and sizes, which can change during the lifespan of the host organism. Because of their unpredictable elemental composition, shape and size, their reactivity and in turn stability are unknown. Therefore, investigation into formation of biogenic NPs and their characterisation requires in-depth analysis of all chemical species of the element by combination of several analytical techniques.

Biosynthesis of HgSe containing particles in the animal tissue was known for some time, but the empirical evidence of the metabolic pathways was missing. An investigation into Hg detoxification in long-finned pilot whales (*Globicephala melas*) using spICP-MS in combination with bio-imaging by synchrotron µ-XRF showed size-dependent changes in the molar composition of biogenic HgSe NPs. While the Hg:Se molar ratio in smaller NPs was < 1, an increase to a molar ratio of 1 was found with growth of the clusters [23]. It was postulated that Hg bound to selenocysteine (SeCys) most probably on the selenoprotein P (SelP) acts as a nucleation centre for binding of additional Hg. Recently, this theory was corroborated by empirical evidence provided by several studies of HgSe formation in seabirds [24, 25]. Using a combination of high-energy resolution X-ray absorption near-edge structure (HRXANES), extended X-ray absorption fine structure (EXAFS) spectroscopy and scanning transmission electron microscopy (STEM), the authors identified mercury-tetraselenolate (Hg(SeR)_4_), an intermediate compound in biosynthesis of HgSe. The authors proposed that Hg(SeR)_4_ is formed via ligand transfer between methylmercury-cysteine (MeHgCys) and SeCys to form MeHgSeCys. During this exergonic reaction, both Hg bonds are cleaved, C-Hg and Hg-S [26], what increases Hg coordination number from two to four leading to a formation of Hg(SeR)_4_. The only selenoprotein which can support formation of Hg(SeR)_4_ is SelP which contains at least 4 SeCys moieties. Therefore, SelP acts as a nucleation centre for biosynthesis of HgSe nanoparticles. The observed size of the HgSe nanocrystals ranged from 5 to 100 nm with the smallest size of 4–5 nm.

Similar observation was made by others, where by means of electron microscopy, HgSe aggregates of 50–100 nm were found in the lysosome of HepG2 cells co-exposed to NaSeO_3_ and HgCl_2_ [27]. The size of the formed aggregates was dose dependent, and the primary HgSe nanoparticles were of 5–10 nm size. It was hypothesised that the SeCys-rich C-terminus of SelP can act as a multinuclear binding centre through natural protein folding and thus can promote the biomineralisation of HgSe through self-assembling what is observed as aggregation or growth of HgSe clusters [23, 25]. Further evidence in support of HgSe nanoparticle formation through Hg(Sec)_4_ intermediate was provided by studies of Hg isotope signatures using multicollector ICP-MS [24, 25, 28–31]. In the published experimental and theoretical studies, the demethylation reaction of MeHg to four-coordinate selenocysteine complex (Hg(Sec)_4_) was reflected in the isotopic differences in δ^202^Hg as a results of mass-dependent fractionation. However, isotopic difference in δ^202^Hg between Hg(Sec)_4_ and HgSe could not be solely explained by mass-dependent fractionation, but a contribution from nuclear volume fractionation was suggested, which could be induced by HgSe biomineralisation.

Biogenic HgSe NPs have been found in a large number of fish and marine mammals available for consumption, but very few studies looked at the potential impact of human exposure to biogenic NPs. A study of HepG2 cells co-exposed to NaSeO_3_ and HgCl_2_ showed that the cells can uptake HgSe naturally formed in the growth media without observed toxic impact [27]. In contrast to the biosynthesised HgSe NPs in the cells, those taken up from the media were stored in the vacuoles rather than in the lysosomes. This observation indicates that cells can distinguish between in situ formed and dietary HgSe NPs. Similarly, rodents have been found to excrete HgSe NPs following a dietary exposure even under Se-deficient conditions which also corroborates non-bioavailability of Se when present in HgSe NPs [32]. Thus, the determination of the dietary exposure to HgSe NPs is not only important to assess the health risk from Hg exposure but also what implication this species has on the source of dietary Se. A recent survey of most consumed fish on Japanese market showed that all analysed fish species contained HgSe NPs, although, based on the measured Hg species, the estimated exposure to particulate Hg was only 0.2% of the provisional tolerable weekly intake [33]. However, the authors were not able to quantify Se in the particulate form, which could provide additional information on the dietary benefits of studied fish species.

Quantification of biogenic Se NPs in Se-rich food and supplements is gaining some interest because not only particulate Se is less toxic than selenite and some organic Se species, but it also can easily be reduced to selenide for biosynthesis of essential organic Se species. A multi-instrumental approach was used to identify the size of Se NPs in two strains of yeast which size varied between a few nm and 250 nm [34]. Intact cells were transported into the plasma of ICP-MS by high nebulisation efficiency introduction system specifically designed for single-cell analysis to determine the selenium content within individual cells. Lysis through mechanical disruption was applied to prove the presence of Se-containing NPs, and released Se NPs down to 20 nm could be detected by spICP-MS. Where spICP-MS reached the size LOD, the complementary information on the NPs < 20 nm was obtained by SDS-containing reversed phase liquid chromatography that allowed to distinguish between NP-bound Se and low-molecular species, probably selenite. In order to check the presence of elemental selenium (the form of Se present in the Se NPs) in the yeast lysates, a derivatisation with sodium sulphite was carried out as well prior to the chromatographic analysis. TEM with energy-dispersive X-ray spectroscopy (EDX) was applied as a confirmative technique.

Similarly, biogenic Se NPs were found in several strains of lactic acid bacteria, bifidobacterial and yeast used in production of dairy products [35]. Location of the Se NPs was strain dependent, either intra or extracellular with one straining containing NPs in both regions. A relatively good agreement was reported between the size of particles detected by spICP-MS and TEM which ranged between 60 and 260 nm. However, when selected bacteria cultures were spiked into the dairy products, the particle number recovery was satisfactory only for one sample out of three although the particle size agreed with measured size without the dairy matrix. This observation highlights the challenges caused by interactions between the NPs and matrix constituents which requires more detailed investigations into sample preparation strategies.

**Fig. 3:**
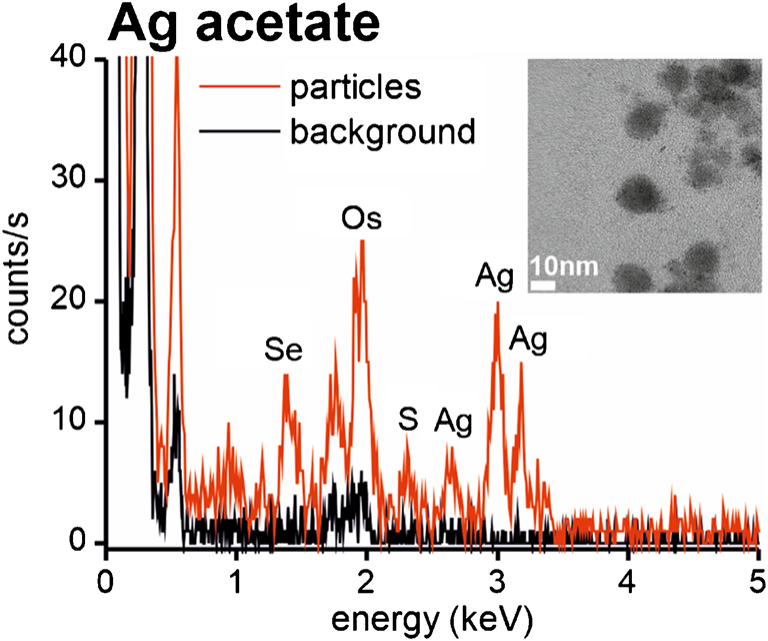
Energy-dispersive x-ray emission spectra of nanosized particles in lysosomes of Ag acetate exposed rats (red line) and corresponding background spectra (black line), i.e. spectra of surrounding tissue containing no visible particles (unstained sections). The osmium signal originated from the tissue fixation procedure in which osmium tetroxide was used as a post-fixative. The TEM image shows the analysed particle-containing area. Originally published in [36]

The anti-microbial properties of Ag NPs led to their wide use in consumer products including food packaging, containers and utensils. Silver NPs are known to be unstable and can undergo partial dissolution and subsequent NP formation. Therefore, a question had arisen, what happens when Ag NPs enter the gastrointestinal tract. Several animal feeding studies provided very good evidence of the dynamic behaviour of Ag NPs in the gastrointestinal tract [36, 37]. Simultaneous exposures of groups of rats to Ag NPs and ionic Ag (Ag^+^) have shown that NPs undergo partial dissolution upon ingestion which is enhanced by the low pH in the stomach; thus, the exposure to NPs could erroneously be considered without a harm. However, the studies have also shown that Ag ions form biogenic NPs in vivo. The biogenic Ag NPs were found in several organs of the studied rats, and it is not clear whether it was the Ag^+^ which were distributed to various organs (GI tract, lungs, testis, brain) and afterwards underwent NPs formation or if the NPs were formed in the GI tract and then re-distributed through the body. Although some elimination of biogenic NPs had been observed, the elimination particularly from the brain and testis occurred at an extremely slow rate. The sizing of NPs was performed by different techniques in each study, and while Loeschner et al. [36] reported size of ≤ 12 nm, NPs detected by van der Zande et al. [37] were between 20 and 30 nm. Elemental analysis of the agglomerated particles by EDX revealed the presence of Se and S [36] (Fig. 3). In another study, biogenic Ag NPs formed during simulated gastrointestinal digestion of AgNO_3_ were found to be associated with S and Cl [38]. A comparison between simulated GI digestion of AgNO_3_ solution with and without protein content found a negligible number of NPs was found in the artificial saliva and gastric juice, but the particle number increased significantly in the artificial intestinal juice. It should be noted that Ag NPs were formed only when proteins were added into the AgNO_3_ solution. The added proteins were to simulate composition of ingested food, and it appears that they provide a nucleation centre for Ag^+^ to bind and eventually form NPs. The size of the NPs was found to be ≤ 20–30 nm which was limited by instruments’ limit of detection. Despite the fact that the deposition of Ag as particles in the human body has been observed and studied for several decades [39], it is only recently that a method for the separation, mass quantification and size characterisation of Ag NPs and Ag^+^ based on SEC–ICP–MS was development and applied to different organs in vivo [40].

Some bio-imaging techniques provide non-destructive analysis and are well suited for exploratory studies of biogenic NPs embedded in the tissue. With particles of unknown reactivity, non-destructive techniques may be preferred as even gentle extraction methods may alter their composition. In a recent study, synchrotron µ-XRF was used to map lead (Pb) and Se distribution pattern in the brain of mice exposed to Pb [41]. The study showed that Pb-exposed mice accumulated significantly more Pb in the brain than the control, and formation of Pb-containing NPs was observed. Interestingly, large number of Pb-containing NPs spatially co-localised with Se NPs and the molar ratio in the large clusters calculated from the elemental maps approached 1. Although no investigation into ionic species of Pb or Se was conducted in this study, the authors have suggested that formation of Pb NPs proceeded through binding to Se in SelP as it was previously demonstrated in biosynthesis of HgSe NPs.

In conclusion, combination of quantitative and qualitative techniques significantly advanced our understanding of formation biogenic NPs. Analysis of both fractions dissolved and particulate are necessary to identify the intermediates between ionic forms of the analyte present in the food and NPs found in the host organisms. Increasing number of publications have found biogenic NPs present in large number of foods; however, very few studies focus on the dietary implications of these food products. Although the interest in the analysis of biogenic NPs is diversifying, a comprehensive analysis is obstructed by their interactions with matrix in which they are present and so far, only semi-quantitative results are being published.

### Outlook

In the last two decades, we have seen a significant increase in the development of analytical methods for the characterisation of NPs. Many of the used techniques, like SEM, TEM, AF4-ICP-MS and spICP-MS, are not part of the classical speciation analysis word; some techniques like (SDS-containing) reversed phase liquid chromatography and SEC coupled to ICP-MS originate from there. Slowly the worlds of NP characterisation and classical speciation are merging, and NPs are no longer considered a separate phenomenon but a part of the trace element species family.

Developments in NP analysis continue, for example, in relation to spICP-MS. ICP-MS and single-particle analysis have been around for several decades, but it is in recent instrument models that the manufacturers provided significantly shorter dwell and settling time and software solutions making the technique accessible to all ICP-MS users. With these fundamental improvements, the interests in spICP-MS applications grew exponentially which resulted in several trends: (i) diversity of analysed food for the presence of NPs expanded; (ii) demands for precise and accurate results increased and with them the need for suitable reference materials as demonstrated by interlaboratory comparison studies [16, 42] and the EU project NanoLyse [43, 44]; (iii) identification of NPs by using the multielement detection capabilities of time-of-flight ICP-MS in single-particle mode is pursued in diverse disciplines of analytical chemistry [45], and it is only a matter of time when it will reach food analysis; and (iv) size detection limits are pushed to lower values by making use of the higher sensitivity of double-focusing or sector field ICP-MS [46, 47]. Food matrices are presenting significant challenges in analysis of NPs and to succeed fundamental research into sample preparation is still required.

Studying the fate of NPs in food and the GIT remains of high importance for assessing the risk related to NPs already present in food and the development of novel foods containing engineered NPs. In vitro dissolution tests are a valuable tool for assessing the bioaccessibility/bioavailability of NPs and could play a potential regulatory role for orally ingested NPs in the future if harmonisation and standardisation efforts are successful. Regarding the analysis of NPs in such complex systems, multi-technique approaches are already applied, but more work needs to be done when it comes to the determination of the “dissolved fraction”. A simplified approach using (ultra)filtration or (ultra)centrifugation might not suffice in a complex matrix containing proteins and other large molecules. Classical speciation analysis like SEC–ICP–MS could be employed to identify and quantify the newly formed species including the matrix-bound ions. Also, the potential of FFF- and chromatography-based techniques coupled to ICP-MS for investigating the NPs and their dissolved species should be further explored.

An increasing variety of biogenic NPs is being identified in food. This class of NPs is currently understudied; however, one can expect steady increase in the number of publications. The extensive research put into identification of intermediates in HgSe NP biosynthesis provided a blueprint for future studies of biogenic NPs and incorporated NP analysis into speciation framework. Investigations into chemical species must include NP analysis to provide comprehensive information about analyte behaviour in the host organism. With the world of NP characterisation and classical speciation growing together, the experts of both worlds should join forces. We might sometimes use a slightly different language, but we can learn a lot from each other.
